# Smoking and Pulmonary Fibrosis: Novel Insights

**DOI:** 10.1155/2011/461439

**Published:** 2011-06-15

**Authors:** Katerina D. Samara, George Margaritopoulos, Athol U. Wells, Nikolaos M. Siafakas, Katerina M. Antoniou

**Affiliations:** ^1^Department of Thoracic Medicine, Faculty of Medicine, University of Crete, 71110 Heraklion, Greece; ^2^Interstitial Lung Disease Unit, Royal Brompton Hospital, London SW3 6NP, UK

## Abstract

The relationship between smoking and pulmonary fibrosis is under debate and intense investigation. The aim of this paper is to review the existing literature and identify further areas of research interest. Recently the negative influence of cigarette smoking on IPF outcome was highlighted, as non-smokers exhibit a better survival than ex-smokers and combined current- and ex-smokers. In patients with non-specific interstitial pneumonia (NSIP), a high prevalence of emphysema was recently demonstrated, providing an indirect support for a smoking pathogenetic hypothesis in NSIP. The coexistence of pulmonary fibrosis and emphysema has been extensively described in a syndrome termed combined pulmonary fibrosis and emphysema (CPFE). Connective tissue disorders (CTDs) are a group of autoimmune diseases which affect the lung, as one of the most common and severe manifestations. However, the relationship between smoking and autoimmune disorders is still conflicting. Rheumatoid arthritis results from the interaction between genetic and environmental factors, while the best established environmental factor is tobacco smoking. Smoking has also a negative impact on the response of the RA patients to treatment. The aforementioned smoking-related implications give rise to further research questions and certainly provide one more important reason for physicians to advocate smoking cessation and smoke-free environment.

## 1. Introduction

Smoking is one of the most prevalent and addictive habits, affecting billions of people and influencing their behaviour. The health risks and effects of tobacco smoking are well known, and their span affects multiple systems of the human body. The direct pathogenetic relationship between cigarette smoking and certain respiratory diseases such as emphysema, chronic obstructive pulmonary disease (COPD), and lung cancer is well documented [1]. Regarding interstitial lung damage there is strong evidence providing links with cigarette smoking. Smoking has been identified as a causative agent in some diffuse parenchymal diseases like respiratory bronchiolitis-interstitial lung disease (RB-ILD) and desquamative interstitial pneumonia (DIP). These diseases have a fairly good prognosis following smoking cessation and treatment [2]. However the relationship between smoking and IPF, the idiopathic interstitial pneumonia with the worst overall prognosis and survival, is under debate, intense research, and investigation. The aim of this paper is to review the existing literature and identify further areas of research interest.

## 2. Idiopathic Interstitial Pneumonias

Recently Katzenstein et al. reported the presence of frequent and often severe fibrosis in cigarette smokers undergoing lobectomy surgery for lung cancer with no clinical evidence of interstitial lung disease [3]. This type of fibrosis is not classifiable by the current criteria for interstitial pneumonias but has distinct histologic features and was termed by the authors as smoking-related interstitial fibrosis (SRIF). SRIF lacks the distinct characteristics of usual interstitial pneumonia (UIP) like honeycomb changes and fibroblastic foci and also lacks significant inflammation that characterises NSIP. What is common in SRIF alongside fibrosis is the presence of emphysema, highlighting the smoking-related origin of this fibrotic phenotype [3]. Complementing the search for smoking-related fibrosis in pathology sections, high-resolution CT (HRCT) has been a useful tool in the study of smoking-related interstitial pneumonias. In a retrospective HRCT study, Marten et al. compared the prevalence and extent of emphysema in smokers with NSIP versus smokers with clinical disease (chronic obstructive pulmonary disease (COPD) and “healthy” smokers [4]. The results revealed emphysema in a striking majority of NSIP cases (77.8%) and COPD cases (73.5%) but not so in the “healthy” smokers (17.5%). The high prevalence of emphysema in the NSIP patients, which did not differ from the COPD controls, provided, according to the authors, an indirect support for a smoking pathogenetic hypothesis in some NSIP patients [4]. The coexistence of pulmonary fibrosis and emphysema was extensively described for the first time in 2005 by Cottin et al. in a syndrome termed combined pulmonary fibrosis and emphysema (CPFE) [5]. Patients with CPFE are mostly older, male, current, or ex-smokers, exhibiting HRCT fibrotic changes such as honeycombing, reticulation and traction bronchiectasis plus emphysema mostly paraseptal. CPFE patients exhibit distinct, mixed patterns of pulmonary function tests compared to IPF and COPD patients; lung volumes remain near normal due to emphysema and hyperinflation, whereas carbon monoxide diffusing capacity is overly decreased and exercise hypoxemia is present. Also the prevalence of pulmonary hypertension (PAH) was very high among CPFE patients (44%) compared to both IPF and COPD patients. The presence of PAH in patients with both pulmonary fibrosis and emphysema is associated with more severe disease course and poor survival, as was also reported by Mejía and colleagues in 2009 [6]. Emphysema and fibrosis in this syndrome are radiographically described to be concurrent and possible genetic or environmental factors that interact with smoking as a trigger are explored [5, 6]. In an experimental mouse model by Lundblad et al. the overexpression of tumor necrosis factor-(TNF-) *α* resulted in major changes in the lungs of the animals, including both fibrosis and emphysematous-like airspace dilatations, thus resembling a possible experimental model of CPFE [7]. 

Although cigarette smoking is believed to be a risk factor for the development of IPF and the disease is known to occur more frequently in smokers [5, 8], there has been some debate regarding possible pathogenetic implications and the effect smoking has on the outcome of the disease. A better survival in current smokers at time of diagnosis with IPF as compared to ex-smokers was reported in 2001 by King Jr. et al. [9]. Possible explanations for the longer survival in patients with IPF who are smoking cigarettes at the time of their initial presentation were that possibly smokers seek attention earlier because of smoking-related symptoms, thus being identified as IPF in a mild stage of the disease. In contrast other smokers attribute their overall symptoms to smoking and postpone medical care until their disease has progressed enough to dictate smoking cessation due to dyspnea. A subsequent study by Antoniou and colleagues also addressed the issue of smoking effect in IPF survival [10]. Smoking-related lung damage and, especially, concurrent emphysema have a major confounding effect in IPF, resulting in preservation of lung volumes and a steep decrease in gas transfer. Moreover, in severe IPF cases, emphysematous regions can be mixed with honeycomb changes—thus the authors used the composite physiologic index (CPI) [11] as a severity variable able to take smoking-related damage independently into account. The authors reported that non-smokers exhibited a better survival and severity-adjusted survival than both ex-smokers and the combined group of current and ex-smokers, thus highlighting the negative influence of cigarette smoking on IPF outcome. The better outcome in the current smokers group when compared to the ex-smokers was attributed to less severe disease at presentation, described as a “healthy smoker effect” [10]. 

Another possible effect of smoking in IPF development, outcome, and response to treatment could be related with the regulation of inflammatory genes. Posttranslational modifications of histone proteins in chromatin structure play a central role in the epigenetic regulation of gene transcription. Histone acetylation and methylation are the two most common and best characterized modifications that function as specific transcription regulators. In interstitial lung disease, the high level of oxidative stress may impair histone deacetylase-(HDAC-) 2 activity, and we know that cigarette smoke induces acetylation of histone H4 and decreases HDAC2 activity and expression [12]. Recently, Coward and colleagues reported that defective histone acetylation is responsible for the repression of the antifibrotic cyclooxygenase-2 gene in IPF [13]. The same group also reported the repression of another antifibrotic gene, the potent angiostatic chemokine gamma interferon-(IFN-*γ*-) inducible protein of 10 kDa (IP-10), in lung fibroblasts from patients with IPF, via histone H3 hypermethylation [14]. More importantly, treatment of diseased cells with HDAC or G9a inhibitors similarly reversed the repressive histone deacetylation and hypermethylation and restored IP-10 expression. These findings strongly suggest that epigenetic dysregulation involving interactions between histone deacetylation and hypermethylation is responsible for targeted repression of IP-10 and potentially other antifibrotic genes in fibrotic lung disease and that this is amenable to therapeutic targeting. Further studies are needed to investigate the relationship between smoking and HDAC dysregulation in IPF, as well as the differences between smokers and non-smokers.

## 3. Connective Tissue Disorders-Associated Interstitial Lung Disease

Tobacco smoking has serious effects on both the innate and the adaptive immunity. It is associated with multiple proinflammatory effects favouring allergic and autoimmune reactions and also immune-suppressive effects predisposing to a lower activity of the immune system against infections. The relationship between smoking and autoimmune disorders is conflicting. Environmental factors and exposures including infections, vaccines, drugs, and smoking have been involved in the complex pathogenesis and clinical development of autoimmune disorders [15]. Connective tissue disorders (CTDs) are a group of autoimmune diseases which affect mainly the joints but frequently present also extra-articular manifestation, with lung involvement being one of the most common. Most of the anatomic compartments of the lung can be affected. Interstitial lung disease associated to CTDs has a prevalence of 15% [16]. Rheumatoid arthritis (RA) is a disorder that results from the interaction between genetic and environmental factors. The best established environmental factor is tobacco smoking whereas the best established genetic factor is HLA-DRB1 shared epitope (SE). Longer duration and severity of smoking increase the risk of RA, which remains high until 10–20 years after smoking cessation [17, 18]. However, it has been demonstrated that both smoking and HLA-DRB1 SE are risk factors exclusively for anticitrulline-positive but not anticitrulline-negative RA and that there is an association of HLA-DR SE with citrulline immunity rather than with RF positivity [19–22]. Recently another gene-environment association has been demonstrated. Polymorphisms of peptidyl-arginine deiminase 4 (PADI4), one of the enzymes that catalyze the citrullination process, highly predispose male smokers to RA, and the genetic heterogeneity observed in the PADI4 polymorphism between populations of Asian and European countries may be partly explained by differences in smoking prevalences among men [23]. Klareskog et al. have observed that smoking is associated with citrullination in the lung by determining the presence of citrullinated proteins in the cells of BAL fluid of smokers (healthy individuals and patients with pulmonary inflammatory conditions) whereas citrullinated proteins were absent in the cells of BAL fluid of nonsmokers [19]. Later, Bongartz et al. have shown that citrullinated proteins are also present in the lung tissue of patients with interstitial pneumonia associated with RA (RA-IP) as well as in patients with idiopathic interstitial pneumonia (IIP), but high antibodies to citrullinated protein antigen (ACPA) titers have been observed only in RA-IP and not in IIP. These findings suggest that citrullination can contribute to the local pathology in ACPA-positive RA patients and that this process is not RA specific but associated with inflammation in certain patients. Interestingly, they found no association between citrullination and smoking status [24]. Smoking has also an impact on the response of the RA patients to treatment. It has been shown that current smokers with early diagnosed RA did not have a good response to biologic agents such as methotrexate and tumor necrosis factor inhibitors compared to former or never-smokers [25]. 

In a very recent study the effect of smoking regarding the respiratory outcome has been evaluated in a cohort of smokers (current, ex) and non smokers with systemic sclerosis (SSc) [26]. The authors have used the Comprehensive Smoking Index (CSI) which integrates components such as smoking intensity, smoking cessation, and time since cessation into a single covariate of smoking effect. They have demonstrated a strong negative correlation of smoking intensity and the FEV1/FVC ratio and a trend towards worsening of DLco. In addition, it was observed that the effect of smoking on DLco is cumulative and almost permanent since DLco continues to decline for 16 years after smoking cessation and only at that time it can start recovering although very slowly. In another study where the impact of smoking in the lung function test pattern has been evaluated, the authors found that, while the restrictive pattern is the most common, the presence of an obstructive element is independent of the smoking status and can be observed also in nonsmokers [27]. Finally, in another recent study, the presence of combined pulmonary fibrosis and emphysema (CPFE) has been evaluated in the context of CTDs [28]. The authors compared patients with CTD and CPFE to patients with CTD without emphysema. Most of the patients of the former group were smokers (current/ex) and had a greater pack-year smoking history, higher lung volumes, lower FEV1/FVC ratio and lower diffusing capacity when compared to the patients of the latter group. The absence of emphysema which is a well-defined smoking-related damage could explain these differences.

## 4. Conclusions

In the past ten years the role of cigarette smoking in the pathogenesis, progression and outcome of interstitial lung disorders, both idiopathic, and secondary, has been elucidated. A number of smoking-related pathogenetic implications are now being established giving rise to further research questions. The interaction of smoking and genetic susceptibility in the pathogenesis of lung fibrosis is multiple and complex. The issue of smoking affecting the patients' response to treatment has to be addressed, in order to provide therapies tailored to different patient groups needs. Furthermore smoking could be associated independently with serum or BAL biomarkers providing insight in the outcome of fibrotic lung diseases. It is important to remember, however, that, apart from investigating smoking-related pathogenetic pathways and other harmful consequences, all physicians have to advocate smoking cessation and a smoke-free environment.
